# Point-of-care diagnostics for rapid determination of prostate cancer biomarker sarcosine: application of disposable potentiometric sensor based on oxide-conductive polymer nanocomposite

**DOI:** 10.1007/s00216-023-04818-0

**Published:** 2023-06-30

**Authors:** Hend Z. Yamani, Nardine Safwat, Amr M. Mahmoud, Miriam F. Ayad, Maha F. Abdel-Ghany, Mohammed M. Gomaa

**Affiliations:** 1 Pharmaceutical Analytical Chemistry Department, Faculty of Pharmacy, Ain Shams University, Cairo, 11566 Egypt; 2 Pharmaceutical Analytical Chemistry Department, Faculty of Pharmacy, Cairo University, Cairo, 11562 Egypt; 3 Solid State Physics Department, National Research Centre, Giza, 12622 Egypt

**Keywords:** Sarcosine ion-selective electrode, Solid contact sensors, Ion-to-electron transducers, Tungsten trioxide nanoparticles, Polyaniline-tungsten trioxide nanocomposite, Point-of-care diagnostics

## Abstract

One of the most important reasons for an increased mortality rate of cancer is late diagnosis. Point-of-care (POC) diagnostic sensors can provide rapid and cost-effective diagnosis and monitoring of cancer biomarkers. Portable, disposable, and sensitive sarcosine solid-contact ion-selective potentiometric sensors (SC-ISEs) were fabricated as POC analyzers for the rapid determination of the prostate cancer biomarker sarcosine. Tungsten trioxide nanoparticles (WO_3_ NPs), polyaniline nanoparticles (PANI NPs), and PANI-WO_3_ nanocomposite were used as ion-to-electron transducers on screen-printed sensors. WO_3_ NPs and PANI-WO_3_ nanocomposite have not been investigated before as ion-to-electron transducer layers in potentiometric SC sensors. The designated sensors were characterized using SEM, XRD, FTIR, UV-VIS spectroscopy, and EIS. The inclusion of WO_3_ and PANI in SC sensors enhanced the transduction at the interface between the screen-printed SC and the ion-selective membrane, offering lower potential drift, a longer lifetime, shorter response time, and better sensitivity. The proposed sarcosine sensors exhibited Nernstian slopes over linear response ranges 10^−3^–10^−7^ M, 10^−3^–10^−8^ M, 10^−5^–10^−9^ M, and 10^−7^–10^−12^ M for control, WO_3_ NPs, PANI NPs, and PANI-WO_3_ nanocomposite-based sensors, respectively. From a comparative point of view between the four sensors, PANI-WO_3_ nanocomposite inclusion offered the lowest potential drift (0.5 mV h^−1^), the longest lifetime (4 months), and the best LOD (9.95 × 10^−13^ M). The proposed sensors were successfully applied to determine sarcosine as a potential prostate cancer biomarker in urine without prior sample treatment steps. The WHO ASSURED criteria for point-of-care diagnostics are met by the proposed sensors.

**Graphical abstract:**

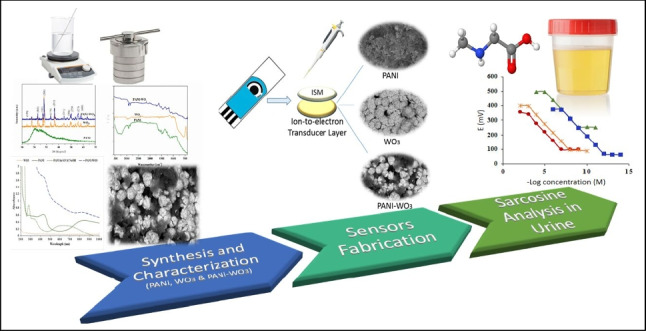

**Supplementary information:**

The online version contains supplementary material available at 10.1007/s00216-023-04818-0.

## Introduction

Point-of-care testing provides rapid analyte detection close to the patient, which facilitates an improved diagnosis, monitoring, and management of different diseases. Currently, the early screening, diagnosis, and monitoring of cancer require expensive, time-consuming, and sophisticated devices that require high expertise personnel and centralized laboratories. For early screening and cancer biomarker detection, new devices are required for point-of-care diagnostics [1–4].

Sarcosine, also known as N-methylglycine, is a byproduct and intermediate in the synthesis and degradation of glycine. It is considered a potential biomarker of prostate cancer [5, 6]. The concentration levels of sarcosine range from 2 to 20 µM in the urine of prostate cancer patients based on the cancer progression state, which may reach the metastasis stage in some cases [7, 8] Moreover, the high levels of sarcosine have been connected to various diseases such as dementia, Alzheimer’s, dimethylglycine dehydrogenase deficiency, sarcosinemia, eosinophilic esophagitis, and Lewy body disease [9]. Different methods have been reported for sarcosine determination, including enzymatic colorimetric or fluorometric assays [10–12], HPLC with fluorescence detection [13], LC-MS [14, 15], GC-MS [16], and capillary electrophoresis [17]. However, these methods have some major disadvantages, such as the need to additional time-consuming sample pre-treatment or derivatization steps, expertise in operating the instruments, and the high cost of enzymes and instrumental setup. To overcome these drawbacks, electrochemical biosensors are considered attractive alternative analytical tools for fabricating point-of-care diagnostics [9]. They are highly selective, sensitive, rapid, inexpensive, and simple to use and handle, which allows early diagnosis and management of diseases, therefore facilitating timely therapy decisions [1].

One of the most important classes of electrochemical sensors is potentiometric sensors that transduce the activity of a certain dissolved analyte into an electrical potential. However, only a few potentiometric sensors based on molecularly imprinted polymers or sarcosine antibody were reported for sarcosine determination [18–20].

The conventional liquid contact sensors, in which the ion-selective membrane (ISM) is inserted between the inner filling and the sample solutions, show certain disadvantages such as fragility, the possibility of the flux of primary ion from the inner filling solution into the sample, and the risk of leakage or drying of inner filling solution. Accordingly, the elimination of inner filling solution in SC sensors overcomes these drawbacks by offering good rigidity, durability, and compatibility with miniaturization [21]. Moreover, screen-printed electrodes show extra advantages over other classical solid-contact electrodes, as being disposable, inexpensive, and versatile. However, SC sensors may suffer from potential drift and a prolonged response time because of the high charge transfer resistance at the interface between ISM and SC. Besides, the water layer formed between the SC and the membrane acts as an electrolyte reservoir which re-equilibrates with each sample composition change leading to a long response time. In addition, diffusion of atmospheric gases as CO_2_ or O_2_ through the ISM is favoured by this aqueous layer. A change in pH at the interface or redox side reactions may occur because of CO_2_ or O_2_, respectively, causing potential drifts. Moreover, the water layer may decrease the sensor lifetime via detachment of the membrane from the SC [22, 23]. To overcome those drawbacks, different electroactive materials were introduced as ion-to-electron transducer layers to be incorporated between the SC and the ISM.

Several electroactive conducting polymers with mixed ionic and electronic conductivities, as poly(3-octylthiophene) (POT), polypyrrole (PPy), and poly(3, 4-ethlyenedioxythiophene) (PEDOT), were reported to be used as ion-to-electron transducers [24]. PANI is one of the most favourable conducting polymers due to its excellent electrical properties, good hydrophobicity and stability, and ease of preparation from a low-cost monomer [25, 26]. Recently, metal oxides have been introduced as ion-to-electron transducers in potentiometric SC sensors [27, 28]. However, the research in this field is still limited and needs further investigation. WO_3_ receives considerable attention in photocatalysis, electrochemistry, and phototherapy due to its high chemical stability, excellent electrical conductivity, and good adherence to the substrate [29]. Among approaches to design a robust sensor is the combination of two types of materials to gain the beneficial properties of both materials into one electrode. Nanocomposite materials have attracted attention and have been widely used in many applications due to their synergistic or complementary properties derived from several components [30].

To the best of our knowledge, WO_3_ NPs and PANI-WO_3_ nanocomposite have not been investigated before as ion-to-electron transducer layers in potentiometric SC sensors. Thus, the present study describes the chemical synthesis, characterization, and incorporation of WO_3_ NPs, PANI NPs, and PANI-WO_3_ nanocomposite as an ion-to-electron transducer in potentiometric SC sensors for the determination of sarcosine in urine. The effect of their incorporation was investigated by comparing it with a control sensor that was designed by excluding the ion-to-electron transducer layer.

## Experimental

### Chemicals and solutions

Sarcosine, glycine, urea, creatinine, sodium tungstate dihydrate, calix-4-arene, high molecular weight polyvinyl chloride (PVC), 2-nitrophenyl octyl ether (*o*-NPOE), sodium tetraphenylborate (NaTPB), and tetrahydrofuran (THF) were obtained from Sigma-Aldrich. Sodium dodecyl sulfate (SDS), aniline, ammonium persulfate, and oxalic acid were purchased from Alfa Aesar. Boric acid, phosphoric acid, acetic acid, hydrochloric acid, and sodium hydroxide were obtained from El-Nasr Company. Sarcosine solutions (1 × 10^−2^ to 1 × 10^−14^ M) were prepared using Britton-Robinson buffer (pH 5.0).

Twenty-four-hour urine samples were freshly obtained from a healthy male volunteer, 35 years old, and were diluted using Britton-Robinson buffer (pH 5.0).

### Instrumentation

Particle size was estimated by dynamic light scattering (DLS) using a Malvern Zetasizer Nano-ZS (Malvern, UK) equipped with a 4-mW He-Ne laser operating at a wavelength of 633 nm. Surface morphology was studied using a Quanta FEG 250 scanning electron microscope (SEM) (FEI, USA) with accelerating voltages ranging from 200 V to 30 kV and magnifications ranging from 20 × to 1,000,000 × . The X-ray diffraction (XRD) analysis was carried out using a PANalytical X’Pert diffractometer using CuKα1 radiation at 45 kV and 40 mA (Malvern, UK). Fourier transform infrared (FTIR) spectra were recorded using a Vertex 80 V FTIR spectrometer (Bruker, Germany). UV-VIS spectrophotometric measurements were performed using a 1601 PC UV-Visible double-beam spectrophotometer (Shimadzu, Japan). To evaluate the electrochemical properties of the developed layers, electrochemical impedance spectroscopy (EIS) was conducted using a potentiostat/galvanostat PGSTAT204 with Nova 11.1.1 software (Metrohm Autolab, Switzerland). A three-electrode configuration was used consisting of a screen-printed sensor with the studied layer, 3-mm diameter (CH Instruments, USA) as the working electrode, an Ag/AgCl electrode (Metrohm, Switzerland) as the reference electrode, and a Pt wire as the counter electrode. Impedance spectra were recorded in 0.1 M KCl over a frequency range from 100 kHz to 100 mHz using an AC amplitude of 50 mV at the open-circuit potential. A digital ion analyzer model no. 3330 (Jenway, UK) with an Orion 900200 Ag/AgCl double junction reference electrode containing 3 M KCl as a filling solution (Thermo Fisher Scientific, USA) was used for potential measurements.

### Synthesis of WO_3_ NPs

WO_3_ nanoparticles were prepared by a hydrothermal approach using 100 mL of an aqueous solution of 0.125 M sodium tungstate dihydrate. Then 3.2 g of oxalic acid was added to the solution with continuous stirring for 30 min. The hydrochloric acid was slowly dropped into the solution with continuous stirring for 15 min to keep the pH of the solution in the range of 1–2. The solution volume was completed up to 250 mL using double distilled water. Seventy milliliters of the prepared solution was transferred into a 100-mL Teflon-lined hydrothermal autoclave. The autoclave was sealed and kept in an oven at a temperature of 180 °C for 21 h, then cooled down to room temperature. Finally, WO_3_ NP powder was filtrated and washed sequentially with distilled water and ethanol several times to remove impurities, then dried at 60 °C.

### Synthesis of PANI NPs

Micellar emulsion chemical polymerization was used for PANI synthesis [31] using SDS as a surfactant. Equimolar quantities (1.3 M) of SDS and aniline were added to 100 mL of water in a thermostated bath at 20 °C with continuous stirring for 1 h. After that, 100 mL of 1.3 M ammonium persulfate was added dropwise. The polymerization process continued for 2.5 h. The dark green dispersion formed was purified using a 12,000-Da dialysis membrane (Sigma-Aldrich) against deionized water for 48 h. Eventually, the dispersion was washed with water and centrifuged at 15,000 rpm for 10 min four times. The collected sediment dried at 60 °C for 24 h.

### Sensor’s fabrication

The dispersions of ion-to-electron transducer layers were prepared as follows:

1. WO_3_ NP dispersion: 10 mg of WO_3_ NPs was dispersed ultrasonically in 1 mL of THF.
2. PANI NP dispersion: 10 mg of PANI NPs was dispersed ultrasonically in 1 mL of THF.
3. PANI-WO_3_ nanocomposite dispersion: 10 mg of PANI NPs and 10 mg of WO_3_ NPs were dispersed ultrasonically in 1 mL of THF.

Ultrasonic dispersion was performed using the ultrasonic bath model CP500D (42–45 kHz) (Crest Ultrasonics, USA) for 30 min. Fifteen microliters of each dispersion was drop-cast separately on a commercial screen-printed electrode of 3-mm diameter (CH Instruments, USA). The solvent was allowed to evaporate before adding 30 µL of the ISM solution (32.82% PVC, 66.60% NPOE, 0.16% NaTPB, and 0.42% calix-4-arene dissolved in 6 mL THF).

For comparison, a control sensor was prepared by applying the ISM directly on the screen-printed electrode without an ion-to-electron transducer layer. The four sensors were preconditioned by immersing in 10^−2^ M sarcosine solution for 24 h.

## Results and discussion

### Material characterization

The mean hydrodynamic particles sizes of the prepared PANI NPs, WO_3_ NPs, and PANI-WO_3_ nanocomposite were measured by DLS using a zetasizer and found to be 158.5 nm, 146.2 nm, and 222.4 nm, respectively.

**Fig. 1:**
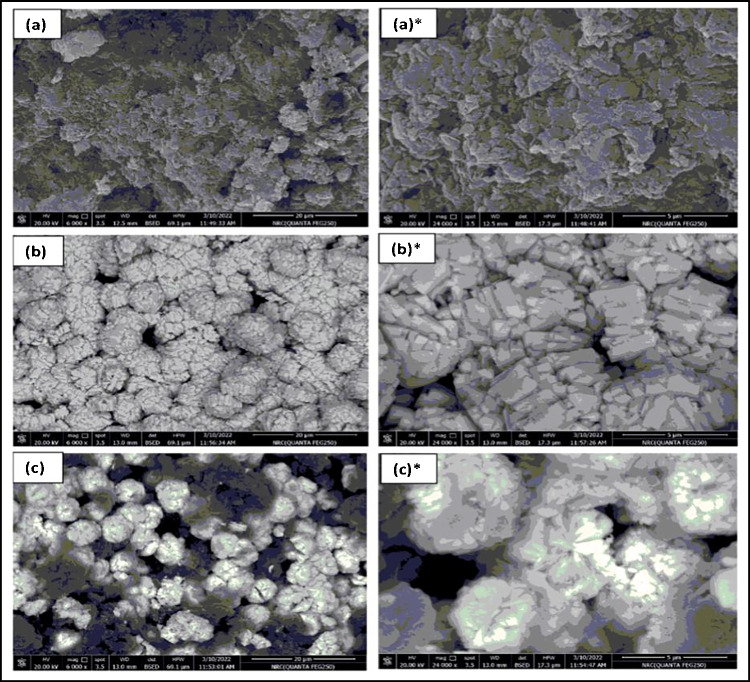
SEM images of PANI NPs (**a**, a*), WO_3_ NPs (**b**, b*), and PANI-WO_3_ nanocomposite (**c**, c*). * Images are of higher magnification

The surface morphology of PANI, WO_3_ NPs, and PANI-WO_3_ layers was investigated by SEM (Fig. 1a–c). The scan of PANI revealed an irregular, rough surface (Fig. 1a, a*). The SEM image of WO_3_ NPs showed a broccoli-like structure that was constructed by several small rectangular nanoplates with a high surface area, as clearly observed in the magnified image (Fig. 1b, b*). The nanocomposite scan illustrated that WO_3_ NPs were embedded and uniformly distributed within the PANI matrix (Fig. 1c, c*). The rough morphology and high surface area can significantly influence signal transduction.

**Fig. 2:**
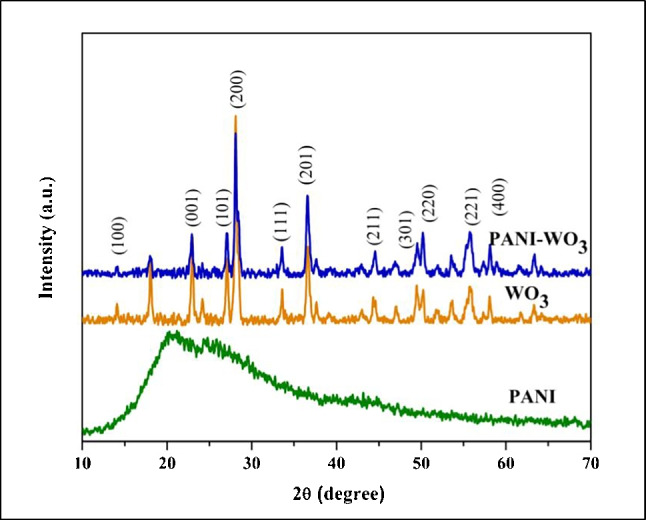
XRD patterns of PANI NPs, WO_3_ NPs, and PANI-WO_3_ nanocomposite

The crystalline structure of PANI, WO_3_, and PANI-WO_3_ nanocomposite was analyzed by XRD as presented in Fig. 2. It can be clearly observed that the XRD patterns of PANI exhibited a broad and low intensity peak at 2θ = 20–30°, which is revealed by its amorphous nature [32]. In contrast, WO_3_ showed sharp crystalline peaks corresponding to the highly crystalline hexagonal structure of the h-WO_3_ phase (JCPDS card no: 00-033-1387) with a preferred orientation (200) plane [33]. On the other hand, the XRD pattern of PANI-WO_3_ nanocomposite shows the same diffraction peaks fitting to the hexagonal h-WO_3_ phase with lower intensities compared to the pure hexagonal h-WO_3_, which might be attributed to the distribution of WO_3_ NPs within the amorphous PANI matrix [32].

**Fig. 3:**
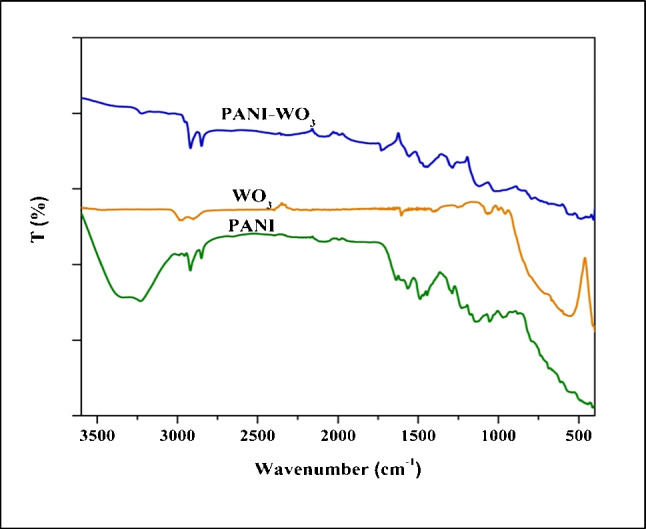
FTIR spectra of PANI NPs, WO_3_ NPs, and PANI-WO_3_ nanocomposite

The FTIR spectra show the in-plane and out-of-plane different mode vibrations of molecules or atoms with associated energies in the IR region (Fig. 3). The main PANI characteristic peaks are assigned as follows: the peaks at 3340 and 3230 cm^−1^ can be attributed to the free (non-hydrogen bonded) N–H stretching vibration and hydrogen-bonded N–H bond between amine and imine sites. The bands at 1565 and 1486 cm^−1^ are related to the C = N and C = C stretching vibration modes for the quinonoid and benzenoid units. The bands at 1290 and 1232 cm^−1^ are assigned to the C–N stretching mode of benzenoid units, while the band at 1112 cm^−1^ to quinonoid unit. The peak at 793 cm^−1^ is attributed to C–H out of plane bending vibrations of the benzenoid unit [34]. In the FTIR of WO_3_, the broad band in the range of 600–800 cm^−1^ represents W-O-W stretching modes in WO_3_ crystal [35]. The FTIR spectrum of PANI-WO_3_ nanocomposite showed the main peaks of pure PANI combined with peaks of WO_3_ with a small shift and lower intensities, indicating the formation of a PANI-WO_3_ matrix as confirmed by XRD measurements.

**Fig. 4:**
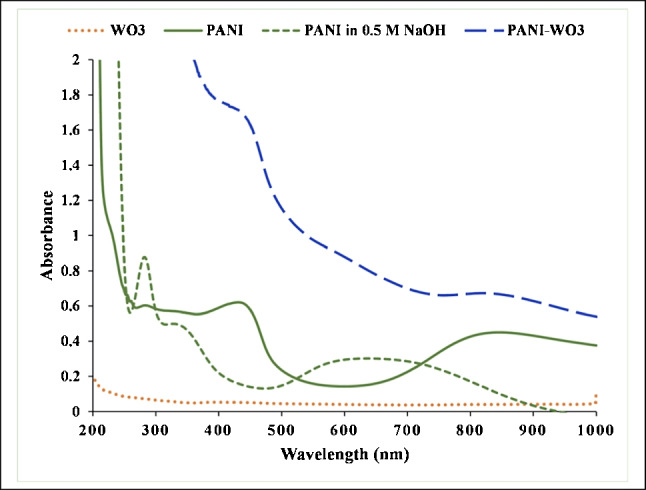
UV-VIS spectra of PANI NPs in the ES form and after dedoping to the EB form using 0.5 M NaOH, WO_3_ NPs, and PANI-WO_3_ nanocomposite

Figure 4 shows the UV-VIS absorption spectrum of PANI dispersion in the conducting form (emeraldine salt). At 785 nm, it revealed localized polaron bands (π—polaron transition). At 420 nm, it showed (polaron—π* transition) and at 350 nm (π – π* band). In alkaline pH, the PANI characteristic absorption spectrum indicates the conversion of PANI NPs from the conducting emeraldine salt to the insulating emeraldine base. A shift from 350 to 320 nm was observed in the polaron band of the π – π*. Moreover, a peak at 560 nm appeared, which is assigned to the exciton band of base form [31]. In the case of PANI-WO_3_ dispersion, the absorption spectrum showed the same characteristic peaks of PANI with higher absorbance due to the introduction of WO_3_ [36].

### Electrochemical characterization

EIS measurements were conducted in 0.1 M KCl solution in the frequency range of 100 kHz to 100 mHz, using Randles’ equivalent circuit (Fig. S1). EIS measurements in the high-frequency region represent bulk membrane resistance (*R*_bc_) in conjunction with contact resistance between the electronic conductor and the ISM. The geometric capacitance value (*C*_g_) can also be obtained from the high-frequency region while the low-frequency region represents double-layer capacitance (*C*_dl_) together with charge-transfer resistance (*R*_ct_) at the interface between the electronic conductor and the ISM. This data provides indications about the ease of ion-to-electron transfer at the electrode interface [37]. As shown in Table S1, the nanocomposite PANI-WO_3_-based sensor showed the lowest *R*_bc_ and *R*_ct_, while its *C*_g_ and *C*_dl_ were the highest. This could be attributed to the synergistic effect of both PANI and WO_3_ NPs when combined together. Besides the outstanding electrochemical properties of the conductive polymer PANI, the addition of WO_3_ NPs with their high surface area, as proved by SEM, and their high capacitance to PANI matrix resulted in a nanocomposite with improved capacitance and lower resistance. As a consequence, this should provide easier ion-to-electron transfer at the electrode interface between the ISM and the underlying SPE with a stable potentiometric response.

### Potentiometric response

**Fig. 5:**
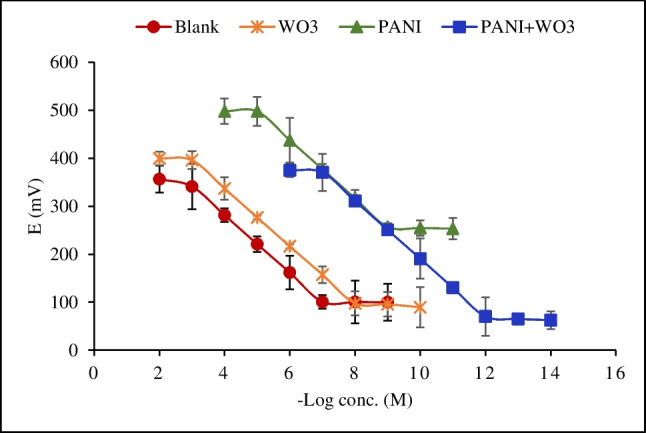
Profile of the potential (mV) versus -log concentrations of sarcosine (M) of the proposed sensors. The standard deviation of three measurements (*n* = 3) is represented by the error bars

The performance of WO_3_ NPs, PANI NPs, and PANI-WO_3_ nanocomposite-based sensors was evaluated compared to a control sensor free from an ion-to-electron transducer layer in batch mode. The potentiometric responses were measured in sarcosine solutions prepared in Britton-Robinson buffer (pH 5.0) in the range of 10^−2^–10^−14^ M. The calibration curves of the four proposed sensors are presented in Fig. 5. The limit of detection (LOD) was calculated from the intersection of the two extrapolated linear segments of the calibration curves. The potentiometric performance characteristics of the four proposed sensors are summarized in Table 1.

**Table 1:** The potentiometric performance characteristics of the four proposed sensors

Parameter	Blank sensor	WO_3_-based sensor	PANI-based sensor	PANI-WO_3_-based sensor
Linear range (M)	10^−3^–10^−7^	10^−3^–10^−8^	10^−5^–10^−9^	10^−7^–10^−12^
LOD (*M*)^a^ ± SD	(9.84 ± 0.1) × 10^−8^	(9.79 ± 0.4) × 10^−9^	(9.54 ± 0.2) × 10^−10^	(9.95 ± 0.1) × 10^−13^
Slope (mV/decade) ± SD^b^	− 60.07 ± 0.3	− 59.76 ± 0.2	− 60.23 ± 0.2	− 60.13 ± 0.1
Intercept (mV) ± SD^b^	521.41 ± 1.7	575.41 ± 1.3	798.69 ± 1.6	791.67 ± 1.4
Correlation coefficient (*r*)	0.9999	0.9999	0.9999	0.9999
Response time (s) ± SD	20 ± 2.5	15 ± 2	10 ± 1.5	15 ± 1
Lifetime (months)	2	2.5	3	4
Potential drift (mV h^−1^) ± SD	10 ± 1	0.7 ± 0.2	0.8 ± 0.2	0.5 ± 0.1

^a^Limit of detection measured by intersection of the two linear extrapolated arms of Fig. 5

^b^*n* = 5 calibrations

From Table 1, it can be clearly seen that the inclusion of WO_3_ NPs, PANI NPs, and PANI-WO_3_ nanocomposite as ion-to-electron transducers improved the performance of the sensors compared to the blank sensor in terms of linearity range, detection limit, response time, lifetime, potential drift, and sensitivity [28, 38, 39].

**Fig. 6:**
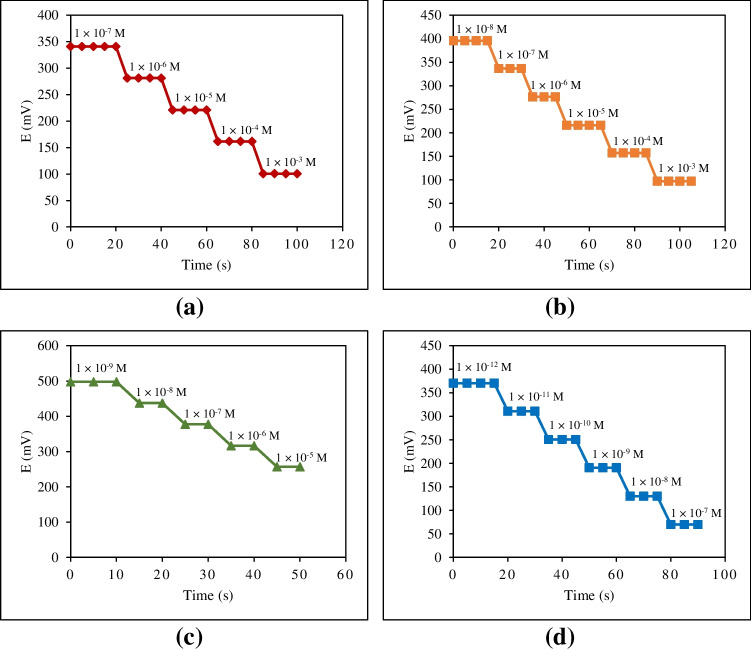
Potential-time curve of the proposed sensors at pH 5.0: **a** blank sensor, **b** WO_3_-based sensor, **c** PANI-based sensor, and **d** PANI-WO_3_-based sensor

The practical response time of the four proposed sensors measured along the linearity range of each sensor at pH 5.0 (Fig. 6).

### Potential stability

To investigate the potential stability of the sensors, the *emf* was recorded over 2 h. A small drift of 0.8, 0.7, and 0.5 mV h^−1^ was obtained for WO_3_, PANI, and PANI-WO_3_ nanocomposite-based sensors, respectively. The blank sensor showed the highest drift (10 mV h^−1^). The improvement in potential stability observed after the addition of WO_3_, PANI, and PANI-WO_3_ nanocomposite is attributed to decreased resistance and increased capacitance, allowing easier ion-to-electron transfer at the sensors interface, which is consistent with EIS results.

The lifetime (long potential stability) of the proposed sensors was found to be 2.5, 3, and 4 months for WO_3_, PANI, and WO_3_-PANI-based sensors, respectively, compared to 2 months for the control sensor.

The longer response time, higher potential drift, and shorter lifetime shown by the control sensor may be attributed to the formation of a water layer between the SC and ISM. In order to evaluate this, a potentiometric water layer test was performed.

### Potentiometric water layer test

The test was performed by measuring the potential drift upon changing from a primary ion solution (sarcosine; 10^−7^ M) to a highly concentrated interfering ion solution (glycine; 10^−3^ M) and then back to the primary ion solution (sarcosine; 10^−7^ M). The water layer that may be formed below the ISM causes the diffusion of gases and ions through the membrane. Changes in pH due to diffusion of CO_2_ and/or diffusion of O_2_ result in redox side reactions that consequently lead to a drift in potential. As the water layer may behave as a reservoir for ions that need re-equilibration upon each change in the analyte composition, longer response times are observed. Besides, the formation of this water layer may cause rapid ISM detachment from the SC, which dramatically affects the sensor lifetime [22, 23].

As shown in Fig. S2, the presence of the proposed ion-to-electron transducer layers improved the stability of sensors and decreased the potential drift to 0.7 mV h^−1^, 0.8 mV h^−1^, and 0.5 mV h^−1^ for WO_3_, PANI, and PANI-WO_3_-based sensors, respectively. However, in the case of the control sensor, where no ion-to-electron transducer layer was used, a layer of water was formed below the ISM resulting in a high potential drift of 10 mV h^−1^. In addition to PANI’s outstanding properties in ion-to-electron transduction, the highly hydrophobic character of PANI could reduce the formation of the aqueous layer [40]. Moreover, the high surface area offered by WO_3_ NPs could provide strong adhesion between the SC and the membrane.

### Effect of pH on sensor performance

The effect of pH on the sensor’s response was investigated using Britton-Robinson buffer over the pH range of 2–11. As shown in Fig. S3, the four sensors showed constant potentials over the pH range of 2–6. Accordingly, this range was chosen as a working pH range for the sensors. The pK_1_ and pK_2_ of sarcosine are 2.98 and 8.55, respectively. Therefore, at different pH values, sarcosine exists in different forms [41]. At acidic pH, sarcosine is mainly in its protonated form, which may enhance attraction with the anionic sites of NaTPB within the PVC membrane. However, at higher pHs, sarcosine molecules start to acquire a negative charge, causing repulsion with tetraphenyl borate and consequently decreasing the response [42].

This data also indicates that the ISM prevents the transition of the pH-sensitive PANI layer from emeraldine salt form to base form. As previously reported by Lindfors and Ivaska [26], this can be explained by the repulsion between OH^−^ ions in the solution and the anionic NaTPB within the membrane. They also reported that bulky doping anions such as SDS or DBSA can suppress PANI pH sensitivity instead of small ones such as Cl^−^ [43].

### Sensor’s selectivity

Urine is an aqueous solution of greater than 95% water, with a minimum of these remaining constituents as urea 9.3 g/L, creatinine 0.670 g/L, chloride 1.87 g/L, sodium 1.17 g/L, and potassium 0.750 g/L. Separate solution method was used to investigate the potentiometric selectivity coefficients of the proposed sensors for structurally related compounds such as glycine. Selectivity towards components that may be present in urine, such as creatinine, urea, uric acid, and proteins (albumin, gamma globulin, and alpha-1-acid glycoprotein), was investigated.

Log ϰ^pot^_sarcosine, glycine_ =  − 0.77, − 3.79, − 2.37, and − 2.00, while Log ϰ^pot^_sarcosine, creatinine_ =  − 1.18, − 4.34, − 2.64, and − 3.50, Log ϰ^pot^_sarcosine, urea_ =  − 0.34, − 3.82,—− 2.86, and − 3.36, Log ϰ^pot^_sarcosine, uric acid_ =  − 1.17, − 4.33, − 2.63, and − 3.49, Log ϰ^pot^_sarcosine, albumin_ =  − 0.33, − 3.79, − 2.83, and − 3.33, Log ϰ^pot^_sarcosine, gamma globulin_ =  − 1.15, − 4.23, − 2.57, and − 3.41, and Log ϰ^pot^_sarcosine, alpha-1-acid glycoprotein_ =  − 1.09, − 4.04, − 2.46, and − 3.26 for control sensor and WO_3_, PANI, and WO_3_-PANI-based sensors, respectively.

The selectivity obtained may be attributed to the selective complexation with the ionophore. The interaction between calixarene host and guest molecules to form an inclusion complex is primarily governed by the size of the calixarene cavity, which determines the selectivity and binding strengths of these receptors to different guest molecules according to size-fit factor. Sarcosine may form an inclusion complex via its N-methyl group deeply embedded in the ionophore cavity. This structural arrangement allows the stabilization of the complex by CH-π contacts between the N-methyl residue and the aromatic pocket, showing higher selectivity over structurally related glycine as reported by Biavardi et al. [44]. Moreover, owing to the lipophilicity of sarcosine compared to hydrophilic ions such as Na^+^, K^+^, and Cl^−^ or the less hydrophobic glycine, they are incapable of replacing sarcosine in the ion-selective membrane.

### Direct determination of sarcosine in urine

For determination of sarcosine in urine using blank, WO_3_-based, and PANI-based sensors, 5 mL of urine was transferred into 50-mL volumetric flasks separately, and different volumes of sarcosine standard solution (10^−2^ M) were added and completed to the volume with Britton-Robinson buffer (pH 5.0). Regarding the PANI-WO_3_-based sensor, a higher dilution ratio is used so that the clinical range of sarcosine in urine would be covered by the concentration range of the proposed sensor. Accordingly, 100 µL of urine was transferred into 50-mL volumetric flasks separately, spiked with different volumes of sarcosine standard solution then completed to the volume with Britton-Robinson buffer (pH 5.0). The potentiometric response was recorded. The percentage recoveries obtained by the proposed sensors are demonstrated in Table S2. The accurate and precise percentage recoveries obtained confirmed the ability of the proposed sensor to be applied for sarcosine determination in urine without the need for preliminary extraction, purification, or derivatization steps.

### Compliance of the PANI-WO_3_ point-of-care (POC) diagnostic sensor with WHO ASSURED criteria

Following the WHO guidelines, our proposed sensor meets the “ASSURED” criteria for POC diagnostics [45] as being: (1) affordable: the screen-printed electrodes as a substrate are an inexpensive platform compared to other costly instrumentations used in other techniques. Moreover, PANI and WO_3_ are synthesized from cheap precursors; (2) sensitive: the PANI-WO_3_-based sensor showed extreme sensitivity with a LOD 9.95 × 10^−13^ M. The other sensors showed also low detection limits of 9.84 × 10^−8^, 9.79 × 10^−9^, and 9.54 × 10^−10^ M for blank, WO_3_, and PANI-based sensors, respectively, which could easily reach sarcosine concentration in urine; (3) selective: the sensor can detect sarcosine quantitatively in the presence of its interferants, which would reduce false positives; (4) user-friendly: the proposed sensors are simple to use; (5) rapid: the sensors showed a rapid response time ranging from 10 to 20 s. Moreover, they were successfully applied for direct sarcosine determination in urine without the need for additional time-consuming sample pre-treatment or derivatization steps; (6) equipment free: on-site data analysis requires only small portable equipment. In contrast to other commonly used techniques that require complicated equipment, (7) deliverable/disposable.

The proposed sensors in this work were compared to the previously reported electrochemical sensors in literature for sarcosine determination as summarized in Table 2. It is worth mentioning that our WO_3_-PANI-based sensor showed superior sensitivity over the other reported sensors with very short response time.

**Table 2:** Comparison of this work with reported sarcosine electrochemical sensors

The electrochemical sensor	Linear range (M)	Sensitivity	LOD (M)	Response time (s)	Life time	Ref
Molecularly imprinted polymer (MIP) sensor prepared with methacryloylamido histidine monomer	1 × 10^−5^–1 × 10^−9^	− 9.3 mV/decade	1.35 × 10^−10^	˂ 120	> 5.5 months	[18]
MIP sensor prepared with methacrylic acid monomer over silica NPs	1 × 10^−5^–1 × 10^−8^	− 27.8 mV/decade	7.8 × 10^−8^	30	5 months	[19]
Nano anti-sarcosine antibody-based sensor	1 × 10^−5^–1 × 10^−9^	Not mentioned	4.75 × 10^−12^	30	4 months	[20]
Nano anti-sarcosine antibody-graphine oxide-based sensor	1 × 10^−7^–1 × 10^−11^	ot mentioned	3.34 × 10^−12^	60	4 months	
Carbon paste electrode (CPE) modified with TiO_2_ NPs in 1-butyl-3-methylimidazolium tetrafluoroborate ionic liquid	Two linear ranges (1 × 10^−3^–1 × 10^−4^) (5 × 10^−3^–1 × 10^−3^)	0.0526 µA/µM	8 × 10^−5^	Not mentioned	Not mentioned	[46]
CPE modified with nano-scaled MIP using methacrylic acid as a functional monomer	1.1 × 10^−3^–5 × 10^−6^	0.0438 µA/µM	0.38 × 10^−6^	180	Not mentioned	[47]
CPE modified with nano-NiMn_2_O_4_	5 × 10^−6^–1 × 10^−8^	20.839 µA/µM	0.38 × 10^−9^	Not mentioned	6 months	[7]
Glassy carbon electrode modified with graphene-chitosan composite and gold-platinum bimetallic nanoparticles in a polypyrrole matrix with riboflavin electrodeposited on the surface	Two linear ranges (3 × 10^−5^–2.5 × 10^−6^) (6 × 10^−4^–3 × 10^−5^)	Two slopes 0.481 µA/µM & 0.105 µA/µM	0.68 × 10^−6^	Not mentioned	1 week	[48]
Au electrode modified with magnetic Fe_3_O_4_ nanoparticles embedded in zeolitic imidazolate framework-8 to support the MIP using methacrylic acid as a functional monomer	1 × 10^−8^–1 × 10^−10^	0.71 µA/pM	0.4 × 10^−10^	Not mentioned	5 weeks	[49]
Carbon screen-printed electrodes modified by immobilization of sarcosine oxidase	1 × 10^−7^–1 × 10^−8^	3.4 × 10^4^ A/mM	16 × 10^−9^	Not mentioned	2 months	[50]
Screen-printed electrode modified with MWCNT/Nafion/Ni(OH)_2_	25 × 10^−6^–3.2 × 10^−6^	1.569 µA/µM	0.96 × 10^−6^	Not mentioned	Can be used for 86 times	[51]
Indium tin oxide–coated glassy electrode modified with ZnO NPs	1 × 10^−7^–5 × 10^−9^	Not mentioned	7.5 × 10^−9^	Not mentioned	8 days	[52]
Glassy carbon electrode modified with sarcosine oxidase immobilized on graphene, chitosan, and silver NPs	1.77 × 10^−4^–1 × 10^−6^	0.0024 µA/µM	1 × 10^−6^	Not mentioned	2 weeks	[53]
Blank sensor	1 × 10^−3^–1 × 10^−7^	− 60.07	9.84 × 10^−8^	20	2 months	This work
WO_3_ NP-based sensor	1 × 10^−3^–1 × 10^−8^	− 59.76	9.79 × 10^−9^	15	2.5 months	
PANI NP-based sensor	1 × 10^−5^–1 × 10^−9^	− 60.23	9.54 × 10^−10^	10	3 months	
PANI-WO_3_ nanocomposite-based sensor	1 × 10^−7^–1 × 10^−12^	− 60.13	9.95 × 10^−13^	15	4 months	

## Conclusion and future directions

Portable, disposable, and cost-effective sarcosine SC ion-selective potentiometric sensors using PANI NPs, WO_3_ NPs, and PANI-WO_3_ nanocomposite as ion-to-electron transducers were fabricated and compared to transducer-free control sensor. For the first time, WO_3_ NPs or PANI-WO_3_ nanocomposite have been used as ion-to-electron transducer layers in potentiometric SC sensors. The inclusion of PANI and WO_3_ in SC sensors enhanced the ion-to-electron transduction at the interface between the screen-printed sensor and the ISM offering more stable potential responses with lower potential drifts, a faster response time, a longer lifetime, and better sensitivity. The proposed sensors exhibited Nernstian slopes over linear response ranges of 10^−3^–10^−7^ M, 10^−3^–10^−8^ M, 10^−5^–10^−9^ M, and 10^−7^–10^−12^ M for blank sensor, WO_3_ NPs, PANI NPs, and PANI-WO_3_ nanocomposite-based sensors, respectively. From a comparative point of view between the four sensors, the inclusion of PANI-WO_3_ nanocomposite offered the lowest potential drift, the longest lifetime (4 months), and the best sensitivity (9.95 × 10^−13^ M). The proposed sensors proved to be capable to determine sarcosine as a potential prostate cancer biomarker in urine without prior sample treatment steps or using expensive and sophisticated instruments. The analysis could be carried out near the patient without the need to transfer samples to central laboratories. Moreover, they are disposable, rapid, cost-effective, and simple to fabricate and use. Therefore, the proposed sensors could be considered good candidates as point-of-care diagnostics for the rapid diagnosis of prostate cancer.

Searching for new electroactive materials as intermediation layers in SC-ISE has drawn attention for many years. The promising properties of the proposed nanocomposite may encourage upcoming research to investigate other combinations of conducting polymers with metal oxides as ion-to-electron transducers. Further work is needed for microfabrication of the proposed sensors to be integrated with an embedded system providing miniaturized detection device for commercial clinical applications.

## Supplementary Information

Below is the link to the electronic supplementary material.

Supplementary file1 (DOCX 539 KB)

## References

[1] Noah NM, Ndangili PM. Current trends of nanobiosensors for point-of-care diagnostics. J Anal Methods Chem. 2019;2019:1–16. 10.1155/2019/2179718. 10.1155/2019/2179718 PMC6925704 31886019

[2] Hayden O, Luppa PB, Min J (2022). Point-of-care testing—new horizons for cross-sectional technologies and decentralized application strategies. Anal Bioanal Chem.

[3] Macovei DG, Irimes MB, Hosu O, Cristea C, Tertis M. Point-of-care electrochemical testing of biomarkers involved in inflammatory and inflammatory-associated medical conditions. Anal Bioanal Chem. 2022;415:6 415:1033–1063. 10.1007/S00216-022-04320-Z. 10.1007/s00216-022-04320-z PMC9472196 36102973

[4] Uludag Y, Narter F, Sağlam E, Köktürk G, Gök MY, Akgün M, Barut S, Budak S (2016). An integrated lab-on-a-chip-based electrochemical biosensor for rapid and sensitive detection of cancer biomarkers. Anal Bioanal Chem.

[5] Sreekumar A, Poisson LM, Rajendiran TM, Khan AP, Cao Q, Yu J, Laxman B, Mehra R, Lonigro RJ, Li Y, Nyati MK, Ahsan A, Kalyana-Sundaram S, Han B, Cao X, Byun J, Omenn GS, Ghosh D, Pennathur S, Alexander DC, Berger A, Shuster JR, Wei JT, Varambally S, Beecher C, Chinnaiyan AM. Metabolomic profiles delineate potential role for sarcosine in prostate cancer progression. Nature 2009;457:7231 457:910–914. 10.1038/nature07762. 10.1038/nature07762 PMC2724746 19212411

[6] Cernei N, Heger Z, Gumulec J, Zitka O, Masarik M, Babula P, Eckschlager T, Stiborova M, Kizek R, Adam V. Sarcosine as a potential prostate cancer biomarker—a review. Int J Mol Sci. 2013;14:13893–13908. 10.3390/IJMS140713893. 10.3390/ijms140713893 PMC3742224 23880848

[7] Rashedi M, Alizadeh T. A novel non-enzymatic sensor for prostate cancer biomarker sensing based on electrocatalytic oxidation of sarcosine at nanostructured NiMn2O4 impregnated carbon paste electrode. Anal Chim Acta. 2021;1186:339121. 10.1016/J.ACA.2021.339121. 10.1016/j.aca.2021.339121 34756269

[8] Cernei N, Zitka O, Ryvolova M, Adam V, Masarik M, Hubalek J, Kizek R (2012). Spectrometric and electrochemical analysis of sarcosine as a potential prostate carcinoma marker. Int J Electrochem Sci.

[9] Pundir CS, Deswal R, Kumar P. Quantitative analysis of sarcosine with special emphasis on biosensors: a review. Biomarkers. 2019;24:415–422. 10.1080/1354750X.2019.1615124. 10.1080/1354750X.2019.1615124 31050554

[10] Yamkamon V, Phakdee B, Yainoy S, Suksrichawalit T, Tatanandana T, Sangkum P, Eiamphungporn W. Development of sarcosine quantification in urine based on enzyme-coupled colorimetric method for prostate cancer diagnosis. EXCLI J. 2018;17:467. 10.17179/EXCLI2018-1245. 10.17179/excli2018-1245 PMC6046622 30034310

[11] Burton C, Gamagedara S, Ma Y (2012). A novel enzymatic technique for determination of sarcosine in urine samples. Anal Methods.

[12] Masumoto M, Ohta S, Nakagawa M, Hiruta Y, Citterio D (2022). Colorimetric paper-based sarcosine assay with improved sensitivity. Anal Bioanal Chem.

[13] Chung TC, te Li C, Kou HS, Wu HL (2015). High-performance liquid chromatographic analysis of sarcosine as a fluorescent levofloxacin derivative. J Chromatogr Sci.

[14] Jiang Y, Cheng X, Wang C, Ma Y (2010). Quantitative determination of sarcosine and related compounds in urinary samples by liquid chromatography with tandem mass spectrometry. Anal Chem.

[15] Burton C, Gamagedara S, Ma Y (2013). Partial enzymatic elimination and quantification of sarcosine from alanine using liquid chromatography-tandem mass spectrometry. Anal Bioanal Chem.

[16] Cavaliere B, MacChione B, Monteleone M, Naccarato A, Sindona G, Tagarelli A (2011). Sarcosine as a marker in prostate cancer progression: a rapid and simple method for its quantification in human urine by solid-phase microextraction-gas chromatography-triple quadrupole mass spectrometry. Anal Bioanal Chem.

[17] Ramezani Z, Safdarian M, Ghadiri AA. Metal-coded hydrogel magnetic molecularly imprinted polymer for preconcentration and cleanup of sarcosine: determination in urine; coupled to on-column capillary electrophoresis. Talanta 2021;230:122309. 10.1016/J.TALANTA.2021.122309. 10.1016/j.talanta.2021.122309 33934774

[18] Özkütük EB, Diltemiz SE, Avcı Ş, Uğurağ D, Aykanat RB, Ersöz A, Say R (2016). Potentiometric sensor fabrication having 2D sarcosine memories and analytical features. Mater Sci Eng, C.

[19] Fernández-Puig S, Lazo-Fraga AR, Korgel BA, Oza G, Dutt A, Vallejo-Becerra V, Valdés-González AC, Chávez-Ramírez AU. Molecularly imprinted polymer-silica nanocomposite based potentiometric sensor for early prostate cancer detection. Mater Lett. 2022;309:131324. 10.1016/J.MATLET.2021.131324.

[20] Altunkök N, Biçen Ünlüer Ö, Birlik Özkütük E, Ersöz A. Development of potentıometrıc bıosensor for dıagnosıs of prostate cancer. Mater Sci Eng B. 2021;263:114789. 10.1016/J.MSEB.2020.114789.

[21] Hu J, Stein A, Bühlmann P (2016). Rational design of all-solid-state ion-selective electrodes and reference electrodes. TrAC Trends Anal Chem.

[22] Fibbioli M, Morf WE, Badertscher M, de Rooij NF, È Pretsch E. Potential drifts of solid-contacted ion-selective electrodes due to zero-current ion fluxes through the sensor membrane. Electroanalysis. 2000;12:1286–1292. 10.1002/1521-4109(200011)12:16%3C1286::AIDELAN1286%3E3.0.CO;2-Q.

[23] Vázquez M, Bobacka J, Ivaska A, Lewenstam A (2002). Influence of oxygen and carbon dioxide on the electrochemical stability of poly(3,4-ethylenedioxythiophene) used as ion-to-electron transducer in all-solid-state ion-selective electrodes. Sens Actuators B Chem.

[24] Wang Y, Liu A, Han Y, Li T (2020). Sensors based on conductive polymers and their composites: a review. Polym Int.

[25] Hussein LA, Magdy N, Yamani HZ (2017). Stable glycopyrronium bromide solid contact ion selective potentiometric sensors using multi-walled carbon nanotubes, polyaniline nanoparticles and polyaniline microparticles as ion-to-electron transducers: a comparative study. Sens Actuators B Chem.

[26] Lindfors T, Ivaska A (2004). Stability of the inner polyaniline solid contact layer in all-solid-state K+-selective electrodes based on plasticized poly(vinyl chloride). Anal Chem.

[27] Zeng X, Qin W (2017). A solid-contact potassium-selective electrode with MoO2 microspheres as ion-to-electron transducer. Anal Chim Acta.

[28] Lenar N, Paczosa-Bator B, Piech R (2019). Ruthenium dioxide nanoparticles as a high-capacity transducer in solid-contact polymer membrane-based pH-selective electrodes. Microchim Acta.

[29] Huang ZF, Song J, Pan L, Zhang X, Wang L, Zou JJ (2015). Tungsten oxides for photocatalysis, electrochemistry, and phototherapy. Adv Mater.

[30] Rajesh AT, Kumar D (2009). Recent progress in the development of nano-structured conducting polymers/nanocomposites for sensor applications. Sens Actuators B Chem.

[31] Moulton SE, Innis PC, Kane-Maguire LAP, Ngamna O, Wallace GG (2004). Polymerisation and characterisation of conducting polyaniline nanoparticle dispersions. Curr Appl Phys.

[32] Li S, Lin P, Zhao L, Wang C, Liu D, Liu F, Sun P, Liang X, Liu F, Yan X, Gao Y, Lu G (2018). The room temperature gas sensor based on Polyaniline@flower-like WO3 nanocomposites and flexible PET substrate for NH3 detection. Sens Actuators B Chem.

[33] Hassani H, Marzbanrad E, Zamani C, Raissi B (2011). Effect of hydrothermal duration on synthesis of WO 3 nanorods. J Mater Sci Mater Electron.

[34] Dey A, De S, De A, De SK (2004). Characterization and dielectric properties of polyaniline-TiO2 nanocomposites. Nanotechnology.

[35] Belardja MS, Djelad H, Lafjah M, Chouli F, Benyoucef A. The influence of the addition of tungsten trioxide nanoparticle size on structure, thermal, and electroactivity properties of hybrid material-reinforced PANI. Colloid Polym Sci. 2020;298:11:145–63. 10.1007/s00396-020-04720-6.

[36] Pawar SG, Patil SL, Chougule MA, Mane AT, Jundale DM, Patil VB (2010). Synthesis and characterization of polyaniline:TiO2 nanocomposites. Int J Polym Mater Polym Biomater.

[37] Bobacka J (1999). Potential stability of all-solid-state ion-selective electrodes using conducting polymers as ion-to-electron transducers. Anal Chem.

[38] Zeng X, Liu Y, Jiang X, Waterhouse GIN, Zhang Z, Yu L. Improving the stability of Pb2+ ion-selective electrodes by using 3D polyaniline nanowire arrays as the inner solid-contact transducer. Electrochim Acta. 2021;384:13414. 10.1016/J.ELECTACTA.2021.138414.

[39] Abdollahzadeh M, Bayatsarmadi B, Vepsäläinen M, Razmjou A, Asadnia M. Highly stable Li+ selective electrode with metal-organic framework as ion-to-electron transducer. Sens Actuators B Chem. 2022;350:130799. 10.1016/J.SNB.2021.130799.

[40] Jiang W, Liu C, Zhao Y, Waterhouse GIN, Zhang Z, Yu L (2019). A solid-contact Pb 2+ -selective electrode based on a hydrophobic polyaniline microfiber film as the ion-to-electron transducer. Synth Met.

[41] Zhou J, Niu X, Yang C, Huo Z, Lu Y, Wang Z, Cui Y, Wang R. Surface action mechanism and planarization effect of sarcosine as an auxiliary complexing agent in copper film chemical mechanical polishing. Appl Surf Sci. 2020;529:147109. 10.1016/J.APSUSC.2020.147109.

[42] Valenti G, Rampazzo E, Biavardi E, Villani E, Fracasso G, Marcaccio M, Bertani F, Ramarli D, Dalcanale E, Paolucci F, Prodi L (2015). An electrochemiluminescence-supramolecular approach to sarcosine detection for early diagnosis of prostate cancer. Faraday Discuss.

[43] Lindfors T, Ivaska A (2002). pH sensitivity of polyaniline and its substituted derivatives. J Electroanal Chem.

[44] Biavardi E, Tudisco C, Maffei F, Motta A, Massera C, Condorelli GG, Dalcanale E (2012). Exclusive recognition of sarcosine in water and urine by a cavitand-functionalized silicon surface. PNAS.

[45] LaBarre P, Boyle D, Hawkins K, Weigl B. Instrument-free nucleic acid amplification assays for global health settings. Sensing technologies for global health, military medicine, disaster response, and environmental monitoring; and biometric technology for human identification VIII. 2011;8029:802902. 10.1117/12.882868

[46] Bahrami H, Mousavi M, Maghsoudi S (2021). Sensitive voltammetric method for rapid determination of sarcosine as a new biomarker for prostate cancer using a TiO2 nanoparticle/ionic liquid modified carbon paste electrode. Russ J Electrochem.

[47] Sheydaei O, Khajehsharifi H, Rajabi HR. Rapid and selective diagnose of sarcosine in urine samples as prostate cancer biomarker by mesoporous imprinted polymeric nanobeads modified electrode. Sens Actuators B Chem. 2020;309:127559. 10.1016/J.SNB.2019.127559.

[48] Liu T, Fu B, Chen J, Li K (2019). An electrochemical sarcosine sensor based on biomimetic recognition. Microchim Acta.

[49] Tang P, Wang Y, He F (2020). Electrochemical sensor based on super-magnetic metal–organic framework@molecularly imprinted polymer for sarcosine detection in urine. J Saudi Chem Soc.

[50] Rebelo TSCR, Pereira CM, Sales MGF, Noronha JP, Costa-Rodrigues J, Silva F, Fernandes MH (2014). Sarcosine oxidase composite screen-printed electrode for sarcosine determination in biological samples. Anal Chim Acta.

[51] de Cássia Mendonça J, da Rocha LR, Capelari TB, Prete MC, Angelis PN, Segatelli MG, Tarley CRT. Design and performance of novel molecularly imprinted biomimetic adsorbent for preconcentration of prostate cancer biomarker coupled to electrochemical determination by using multi-walled carbon nanotubes/Nafion®/Ni(OH)2-modified screen-printed electrode. J Electroanal Chem. 2020;878:114582. 10.1016/J.JELECHEM.2020.114582.

[52] Selvaraj S, Varshini KS, Sonia T, Jeyaprakash BG, Balamurugan D (2021). Spray deposited ZnO nanograins for enzyme-free detection of sarcosine. Sens Imaging.

[53] Zhou Y, Yin H, Meng X, Xu Z, Fu Y, Ai S (2012). Direct electrochemistry of sarcosine oxidase on graphene, chitosan and silver nanoparticles modified glassy carbon electrode and its biosensing for hydrogen peroxide. Electrochim Acta.

