# Supplementary material for: Point-of-care diagnostics for rapid determination of prostate cancer biomarker sarcosine: application of disposable potentiometric sensor based on oxide-conductive polymer nanocomposite
Source: Anal Bioanal Chem. 2023 Jun 30;415(22):5451–62. doi: 10.1007/s00216-023-04818-0 (PMC10444660; doi:10.1007/s00216-023-04818-0)
Supplement: Supplementary file 1 — Supplementary file1 (DOCX 539 KB) [file 216_2023_4818_MOESM1_ESM.docx]

**Supplementary Material**

**for**

### **Point-of-care diagnostics for rapid determination of prostate cancer biomarker sarcosine: application of disposable potentiometric sensor based on oxide-conductive polymer nanocomposite**

### Hend Z. Yamani^a^ , Nardine Safwat^a*^, Amr M. Mahmoud ^b*^, Miriam F. Ayad^a^, Maha F. Abdel-Ghany^a^, Mohammed M. Gomaa^c^

*^a^* *Pharmaceutical Analytical Chemistry Department, Faculty of Pharmacy, Ain Shams University, Cairo 11566, Egypt*

*^b^* *Pharmaceutical Analytical Chemistry Department, Faculty of Pharmacy, Cairo University, Cairo 11562, Egypt*

*^c^ Solid State Physics Department, National Research Centre, Giza 12622, Egypt*

*Corresponding Authors emails: nardine.safwat2@pharma.asu.edu.eg, [amr.bekhet@pharma.cu.edu.eg](mailto:amr.bekhet@pharma.cu.edu.eg)

**Table S1.** EIS parameters for the proposed electrodes by using the equivalent circuit

| The proposed sensor | R_bc_  (Ω) | C_g_  (nF) | R_ct_  (Ω) | C_d_  (pF) |
| --- | --- | --- | --- | --- |
| WO_3_ based sensor | 119272 | 354 | 293155 | 87.3 |
| PANI based sensor | 70388 | 20.8 | 292551 | 51.7 |
| PANI-WO_3_ based sensor | 34649 | 1624 | 110850 | 147 |

R_bc_ is the bulk membrane resistance together with contact resistance between the ISM and the underlying conductor; C_g_ is the geometric capacitance; R_ct_ is the charge-transfer resistance; C_dl_ is the double-layer capacitance

**Table S2.** Determination of sarcosine in urine by the proposed sensor

| Sensor | Spiked concentration (M) | Recovery % (Mean ± SD)^a^ |
| --- | --- | --- |
| Control sensor | 1×10^-7^ | 101.47 ± 0.01 |
|  | 1×10^-6^ | 98.17 ± 0.03 |
|  | 1×10^-5^ | 98.77 ± 0.02 |
| WO_3_ based sensor | 1×10^-8^ | 97.99 ± 0.01 |
|  | 1×10^-7^ | 99.36 ± 0.01 |
|  | 1×10^-6^ | 97.03 ± 0.002 |
| PANI based sensor | 1×10^-8^ | 96.81 ± 0.01 |
|  | 1×10^-7^ | 100.21 ± 0.01 |
|  | 1×10^-6^ | 96.78 ± 0.02 |
| PANI-WO_3_ based sensor | 1×10^-9^ | 101.06 ± 0.008 |
|  | 1×10^-8^ | 99.79 ± 0.009 |
|  | 4×10^-8^ | 100.43 ± 0.01 |

^a^ Average of three determinations





**Fig S1.** Nyquist plots for impedance spectra recorded in 0.1 M KCl at the open-circuit potential for the proposed sensors (A), Randles equivalent electrical circuit model (B).

**A**

**A**

**B**

**Fig. S2.** Potentiometric water layer test. EMF was recorded successively in (A) 10^-7^ M sarcosine, (B) 10^-3^ M glycine then back to (A) 10^-7^ sarcosine by the proposed sensors


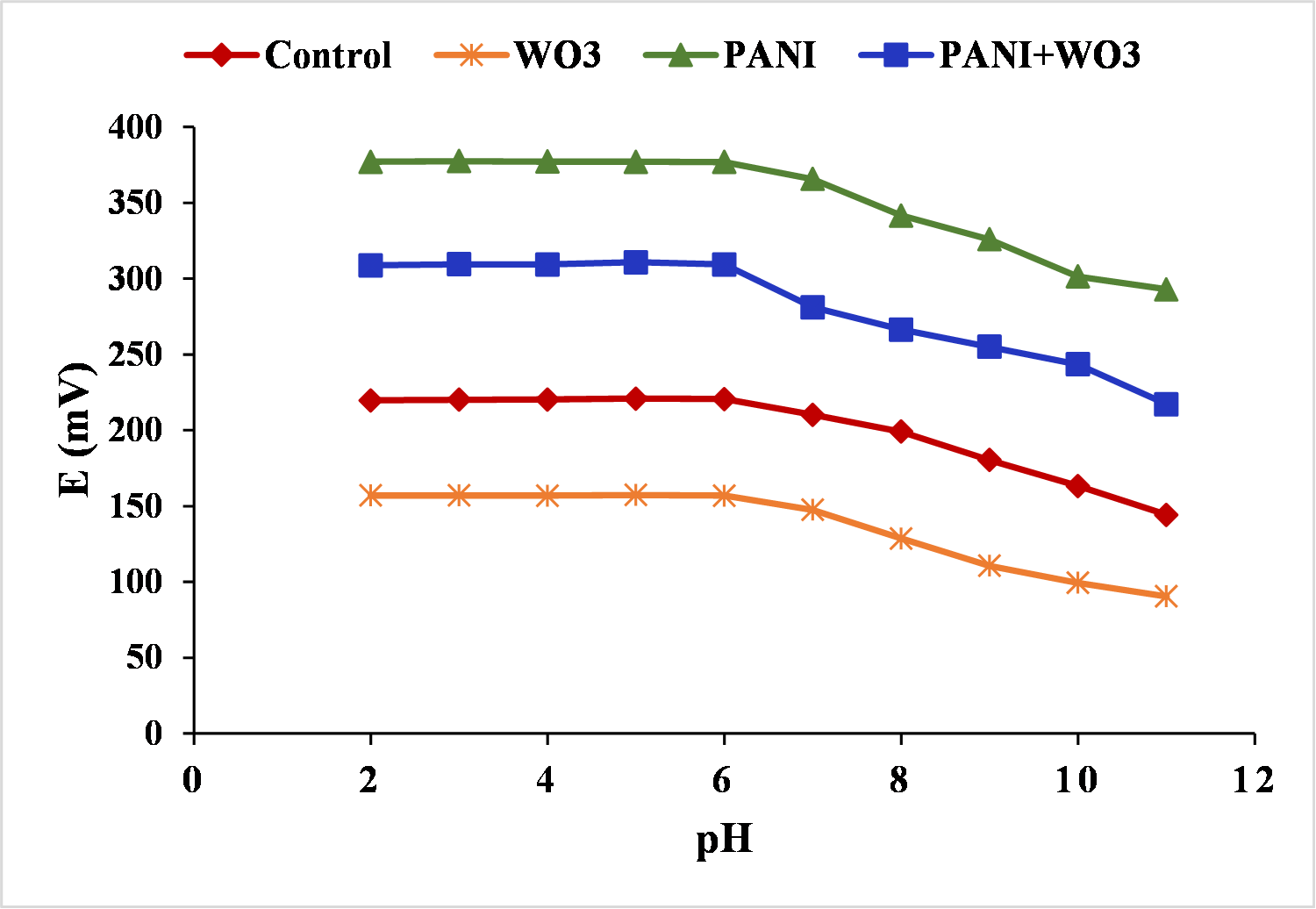


**Fig. S3.** Effect of pH on the response of the proposed sensors.

Effect of pH was studied using 10^-5^ M, 10^-7^ M, 10^-7^ M and 10^-8^ M sarcosine with blank SPE, PANI NPs, WO_3_ NPs and PANI-WO_3_ nanocomposite-based sensors, respectively.
